# The Impact of Renal Denervation on the Progression of Heart Failure in a Canine Model Induced by Right Ventricular Rapid Pacing

**DOI:** 10.3389/fphys.2019.01625

**Published:** 2020-01-31

**Authors:** Wei-Jie Chen, Hang Liu, Zi-Hao Wang, Chang Liu, Jin-Qi Fan, Zheng-Long Wang, Yan-Ping Xu, Bo Zhang, Laxman Gyawali, Qiang Li, Zhi-Yu Ling, Yue-Hui Yin

**Affiliations:** ^1^Department of Cardiology, The Second Affiliated Hospital of Chongqing Medical University, Chongqing, China; ^2^Department of Cardiology, The Affiliated Hospital of Zunyi Medical College, Zunyi, China; ^3^Department of Cardiology, The People’s Hospital of Chongqing Nanchuan District, Chongqing, China

**Keywords:** renal denervation, heart failure, renin–angiotensin system, central sympathetic activation regulation, cardiac remodeling

## Abstract

Heart failure (HF) has been proposed as a potential indication of renal denervation (RDN). However, the mechanisms enabling RDN to attenuate HF are not well understood, especially the central effects of RDN. The aim of this study was to decipher the mode of operation of RDN in the treatment of HF using a canine model of right ventricular rapid pacing-induced HF. Accordingly, 24 Chinese Kunming dogs were randomly grouped to receive sham procedure (sham-operated group), bilateral RDN (RDN group), rapid pacing to induce HF (HF-control group), and bilateral RDN plus rapid pacing (RDN + HF group). Echocardiography, plasma brain natriuretic peptide (BNP), and norepinephrine (NE) concentrations of randomized dogs were measured at baseline and 4 weeks after interventions, followed by histological and molecular analyses. Twenty dogs completed the research successfully and were enrolled for data analyses. Results showed that the average optical density of renal efferent and afferent nerves were significantly lower in the RDN and RDN + HF groups than in the sham-operated group, with a significant reduction of renal NE concentration. Rapid pacing in the RDN + HF and HF-control groups, compared with the sham-operated group, induced a significant increase in left ventricular end-diastolic volume and decrease in left ventricular ejection fraction and correspondingly resulted in cardiac fibrosis and dysfunction. Cardiac fibrosis evaluated by Masson’s trichrome staining and the expression of transforming growth factor-β1 (TGF-β1) were significantly higher in the HF-control group than in the sham-operated group, which were remarkably attenuated by the application of the RDN technique in the RDN + HF group. In terms of central renin–angiotensin system (RAS), the expression of angiotensin II (AngII)/angiotensin-converting enzyme (ACE)/AngII type 1 receptor (AT1R) in the hypothalamus of dogs in the HF-control group, compared with the sham-operated group, was upregulated and that of the angiotensin-(1-7) [Ang-(1-7)]/ACE2 was downregulated. Furthermore, both of them were significantly attenuated by the RDN therapy in the RDN + HF group. In conclusion, the RDN technique could damage renal nerves and suppress the cardiac remodeling procedure in canine with HF while concomitantly attenuating the overactivity of central RAS in the hypothalamus.

## Introduction

Renal denervation (RDN) has been proposed to treat resistant hypertension by damaging the afferent sensory and efferent sympathetic renal nerves (Schlaich et al., 2012; Mahfoud et al., 2013). Alongside, heart failure (HF) had also been proposed to be an indication for RDN (Böhm et al., 2014). The safety of RDN in patients with systolic HF has been demonstrated by previous published pilot studies, which have also indicated that the RDN technique can improve the symptoms, exercise tolerance, and quality of life in patients with HF (Davies et al., 2013; Chen et al., 2016a). However, the efficacy of RDN in patients with HF, as determined through various studies, has been inconsistent (Davies et al., 2013; Chen et al., 2016a; Hopper et al., 2017). Moreover, detailed mechanisms underlying the implication of RDN in the treatment of HF require further investigation.

Excessive activation of the sympathetic nervous system (SNS) and renin–angiotensin system (RAS) plays a crucial role in the progression and aggravation of HF (Böhm et al., 2014). Importantly, cardiac and renal dysfunctions are inextricably intertwined in patients with HF. HF is characterized by global sympathoexcitation, especially to the heart and kidney (Booth et al., 2015; Schiller et al., 2015). It has been demonstrated that the increased renal efferent sympathetic nerve activity induces renal vasoconstriction, decreases renal blood flow with a reduction in glomerular filtration rate, and increases sodium and water reabsorption and renal fibrosis (Booth et al., 2015; Schiller et al., 2015). These alterations in the renal efferent sympathetic nerve also lead to a significant increase in cardiac preload and activation of RAS, which finally results in a continuous progression of cardiac remodeling and exacerbated HF (Booth et al., 2015; Schiller et al., 2015). By damaging the renal efferent sympathetic nerves, it has been proved in animal experiments that the RDN technique improves renal blood flow and renal vascular resistance (Clayton et al., 2011; Schiller et al., 2015), suppresses the over-activation of RAS (Clayton et al., 2011), inhibits renal neprilysin activity (Polhemus et al., 2017), and increases sodium and water excretion (DiBona and Sawin, 1991; Villarreal et al., 1994; Booth et al., 2015).

However, the role of renal afferent nerves in the effects of the RDN procedure remains largely unexplored. Reduction in renal blood flow, which was induced by excessive activation of renal sympathetic nerves in patients with HF, has been shown to activate the renal afferent nerves involved in the regulation of central sympathetic outflow to the heart and peripheral circulation system (Calaresu and Ciriello, 1981; Xu et al., 2012; Booth et al., 2015; Xu et al., 2015). It has been shown that central RAS plays a crucial role in the regulation of central sympathetic outflow (Fujisawa et al., 2011; Zucker et al., 2014). The RDN technique has been shown to reduce the whole-body noradrenaline spillover (Schlaich et al., 2009) and muscle sympathetic nerve activity (MSNA) (Hering et al., 2013). Hence, it may be speculated that the RDN procedure is involved in the central regulatory mechanisms of sympathetic outflow to muscles and peripheral circulation system, majorly by damaging the renal afferent nerves (Booth et al., 2015; Schiller et al., 2015; Chen et al., 2016b). However, this hypothesis has not been directly investigated, and the detailed impact of RDN on the regulation of central sympathetic outflow is also not well characterized. Therefore, we designed an animal study to directly investigate the effect of RDN on renal nerves, the central regulating mechanisms of sympathetic output, and the resulting cardiac structure and function in canines with pacing-induced HF.

## Methods

### Materials and Methods

The detailed materials and methods are presented in the Supplementary Material. In brief, Chinese Kunming dogs were utilized in this study. The animals were obtained from Chengdu Chinese Kunming Dogs Breeding Base in Sichuan Province in China and were kept on standard food in the Laboratory Animal Center of Chongqing Medical University. The experimental protocols of this study were reviewed and approved by the Animal Research Ethics Committee of Chongqing Medical University, following the guidelines of the National Institutes of Health and the Declaration of Helsinki for the Care and Use of Laboratory Animals.

A total of 24 Chinese Kunming dogs were enrolled and randomly assigned into four groups: sham-operated group (*n* = 6), RDN group (*n* = 6), RDN + HF group (*n* = 6), and HF-control group (*n* = 6). The dogs in the RDN group received renal artery angiography and catheter-based RDN using a 6F open-irrigated ablation catheter (AquaSense, Synaptic Medical Limited, Beijing, China). The dogs in the sham-operated group were given the renal artery angiography followed by the placement of ablation catheter without radiofrequency (RF) energy delivery. For the dogs in the RDN + HF group, a high-rate cardiac pacemaker (Fudan University, Shanghai, China) attaching a ventricular endocardial pacing electrode (St. Jude Medical, Inc.) was implanted with continuous right ventricular pacing at 250 beats/min plus the renal artery angiography and catheter-based RDN procedures. The dogs in the HF-control group simultaneously received continuous right ventricular pacing at 250 beats/min as well as the renal artery angiography and placement of ablation catheter without energy delivery.

Transthoracic echocardiographic examinations, surface electrocardiogram (ECG), invasive evaluation of femoral artery pressure, measurements of plasma brain natriuretic peptide (BNP) and norepinephrine (NE) concentrations, and the circulating levels of angiotensin II (AngII) and aldosterone were performed at baseline and 4 weeks after the indicated interventions.

Catheter-based RDN was performed from distal to proximal lumen of the renal artery trunk by point to point burns, both longitudinally and rotationally, using the 6F open-irrigated ablation catheter as described earlier (Lu et al., 2015). Eight to twelve lesions were created in each renal artery as per its length. The temperature was set to 45°C, with 10 W of RF energy and 70-s duration for each lesion. Saline was irrigated at 3 ml/min to cool down the temperature of the tissue–electrode interface during RF energy delivery using the Vation-Cool Pump (Sichuan Jinjiang Electronic Science and Technology Corporation, Chengdu, China).

At the end point of the experiment, dogs were euthanized with an overdose of sodium pentobarbital (200 mg/kg). The renal artery, kidney, brain tissue, left stellate ganglion (LSG), and heart were harvested immediately. Slices of renal artery were stained with hematoxylin–eosin and Masson’s trichrome stains to locate the lesion produced by RDN, which were also stained with polyclonal antibodies to tyrosine hydroxylase (TH; AB117112, Abcam, used at 1:1,000) and calcitonin gene-related peptide (CGRP; no. 250602, Abbiotec, used at 1:100) to assess the damage induced by RDN on the renal efferent sympathetic and afferent sensory nerves. The immunoreactivity and protein expression of TH or CGRP in renal nerve bundles were analyzed and quantified on the basis of the integrated optical density (IOD) of the TH/CGRP-positive nerves using computerized image analysis system (Image-Pro Plus 6.0, Media Cybernetics, Inc., United States). Data are presented as mean IOD of TH/CGRP-positive nerves per unit area of nerve bundle, where the area of nerve bundle was also measured by Image-Pro Plus and expressed as pixels.

Slices of LSG were stained with TH (AB117112, Abcam, used at 1:1,000) to assess the sympathetic activity of LSG and indirectly evaluate the central sympathetic outflow to the heart. The immunoreactivity and protein expression of TH in LSG were also analyzed and quantified on the basis of the IOD of the TH-positive nerves using Image-Pro Plus software.

Slices of interventricular septum tissue were stained with Masson’s trichrome stain to assess the influence of RDN on cardiac fibrosis. Collagen volume fraction was used to evaluate the extents of cardiac fibrosis.

The mRNA and protein expressions of angiotensin-converting enzyme (ACE), AngII type 1 receptor (AT1R), and ACE2 in the hypothalamus were analyzed by real-time reverse transcription–polymerase chain reaction (RT-PCR) and Western blot (WB) techniques, respectively. The primer sequences of all the genes are presented in Supplementary Table S1. The TH protein in the LSG, and also the levels of TH protein and transforming growth factor-β1 (TGF-β1) in the left ventricle, was assayed by WB, whereas the mRNA expression of the beta-1-adrenergic receptor (β1-AR) in the left ventricle was analyzed by quantitative RT-PCR.

The AngII and angiotensin-(1-7) [Ang-(1-7)] levels in the hypothalamus; AngII and aldosterone levels in plasma; BNP and NE concentrations in plasma; and NE concentrations in the kidney and ventricle were detected by enzyme-linked immunosorbent assay (ELISA).

### Statistics

Data were expressed as mean ± SD for continuous variables. BP values were the averages of continuous 10-s beat-to-beat measurements, not any single beat monitoring value, to attenuate the possible interferences from anesthesia and beat-to-beat variations. The differences of continuous variables at baseline and 4 weeks after interventions within the group were analyzed with the use of paired *t*-test. Under the homogeneity of variances, the differences of variables among four groups were analyzed using one-way ANOVA, followed by *post hoc* analysis with least significant difference (LSD) *t*-test for multiple comparisons. However, if homogeneity of variances was violated, the differences of variables among the four groups were analyzed using Welch’s ANOVA, followed by *post hoc* analysis with Games–Howell test. Two-sided *p* < 0.05 was defined as statistically significant. All statistical analyses were performed with SPSS statistical software (version 17.0, Chicago, IL, United States).

## Results

### Animal Survival

During the course of this study, no dogs were excluded owing to renal artery anatomic abnormalities. However, four enrolled dogs died accidentally before completing the follow-up items (independent causes for accidental death are described in the Supplementary Material). Thus, in total, 20 dogs (six in the sham-operated group, five in the RDN group, four in the RDN + HF group, and five in the HF-control group) successfully completed all the research items and were used for data analyses.

### Evaluation of Cardiac Structure and Function

The parameters used to evaluate cardiac structure and function, including results of the transthoracic echocardiographic examination, plasma BNP measurement, circulating AngII and aldosterone and NE levels, and hemodynamic analysis, are presented as mean ± SD in Table 1. There were no significant differences in various parameters at baseline among the different groups, *viz*., left ventricular end-diastolic volume (LVEDV), left ventricular end-systolic volume (LVESV), left ventricular ejection fraction (LVEF), BNP, systolic blood pressure (SBP), and diastolic blood pressure (DBP).

**TABLE 1:** Results of echo parameters, blood pressure, plasma BNP, and circulating AngII and aldosterone and NE levels measured at baseline and 4 weeks after interventions.

Variables	Time	Sham (*n* = 6)	RDN (*n* = 5)	RDN + HF (*n* = 4)	HF (*n* = 5)	†*P*
LVEDV (ml)	Baseline	25.5 ± 6.95	27.4 ± 6.43	27.5 ± 5.50	28.2 ± 6.50	0.933
	4 weeks	28.3 ± 7.93	28.8 ± 4.92	51.3 ± 5.74*	69.0 ± 4.74*	<0.001
LVESV (ml)	Baseline	11.5 ± 2.89	12.8 ± 3.27	12.0 ± 2.16	12.6 ± 2.41	0.891
	4 weeks	12.3 ± 2.75	13.0 ± 2.55	29.0 ± 2.45*	48.8 ± 2.30*	<0.001
LVEF (%)	Baseline	54.5 ± 3.87	54.2 ± 4.82	56.0 ± 5.23	54.6 ± 7.33	0.966
	4 weeks	56.0 ± 3.74	54.8 ± 3.11	43.3 ± 3.50*	29.2 ± 7.46*	<0.001
SBP (mmHg)	Baseline	175.4 ± 20.8	177.6 ± 24.2	172.7 ± 20.7	173.8 ± 23.6	0.989
	4 weeks	179.3 ± 23.0	155.0 ± 18.1*	138.1 ± 25.6*	124.2 ± 15.6*	0.008
DBP (mmHg)	Baseline	118.9 ± 18.4	120.5 ± 19.9	122.3 ± 20.4	119.8 ± 22.2	0.996
	4 weeks	123.6 ± 18.7	110.3 ± 14.7*	96.4 ± 22.6*	88.1 ± 10.5*	0.033
BNP (pg/ml)	Baseline	14.0 ± 5.16	15.4 ± 5.46	13.5 ± 3.42	15.0 ± 6.25	0.945
	4 weeks	14.8 ± 2.50	14.2 ± 3.56	167.3 ± 58.9*	322.6 ± 72.4*	<0.001
AngII (pg/ml)	Baseline	45.6 ± 13.1	52.5 ± 18.4	49.7 ± 16.7	49.8 ± 15.5	0.910
	4 weeks	53.0 ± 17.1	41.2 ± 10.5	170.2 ± 39.0*	299.6 ± 62.1*	<0.001
Ald (pg/ml)	Baseline	472.8 ± 144.8	546.7 ± 137.7	528.7 ± 114.0	536.8 ± 150.2	0.811
	4 weeks	521.2 ± 153.8	497.6 ± 122.0	1,445.5 ± 316.2*	3,026.4 ± 394.5*	<0.001
NE (pg/ml)	Baseline	70.19 ± 22.97	82.45 ± 30.88	67.25 ± 29.08	73.21 ± 32.16	0.874
	4 weeks	75.42 ± 21.56	27.38 ± 9.99	347.23 ± 81.73*	717.19 ± 179.55*	<0.001

Values are represented as mean ± SD. LVEDV, left ventricular end-diastolic volume; LVESV, left ventricular end-systolic volume; LVEF, left ventricular ejection fraction; SBP, systolic blood pressure; DBP, diastolic blood pressure; BNP, brain natriuretic peptide; AngII, angiotensin II; Ald, aldosterone; NE, norepinephrine. * *p* < 0.05, 4 weeks vs. baseline. †*P* indicates the comparison of variables among groups using one-way ANOVA. See text for further description.

**FIGURE 1:**
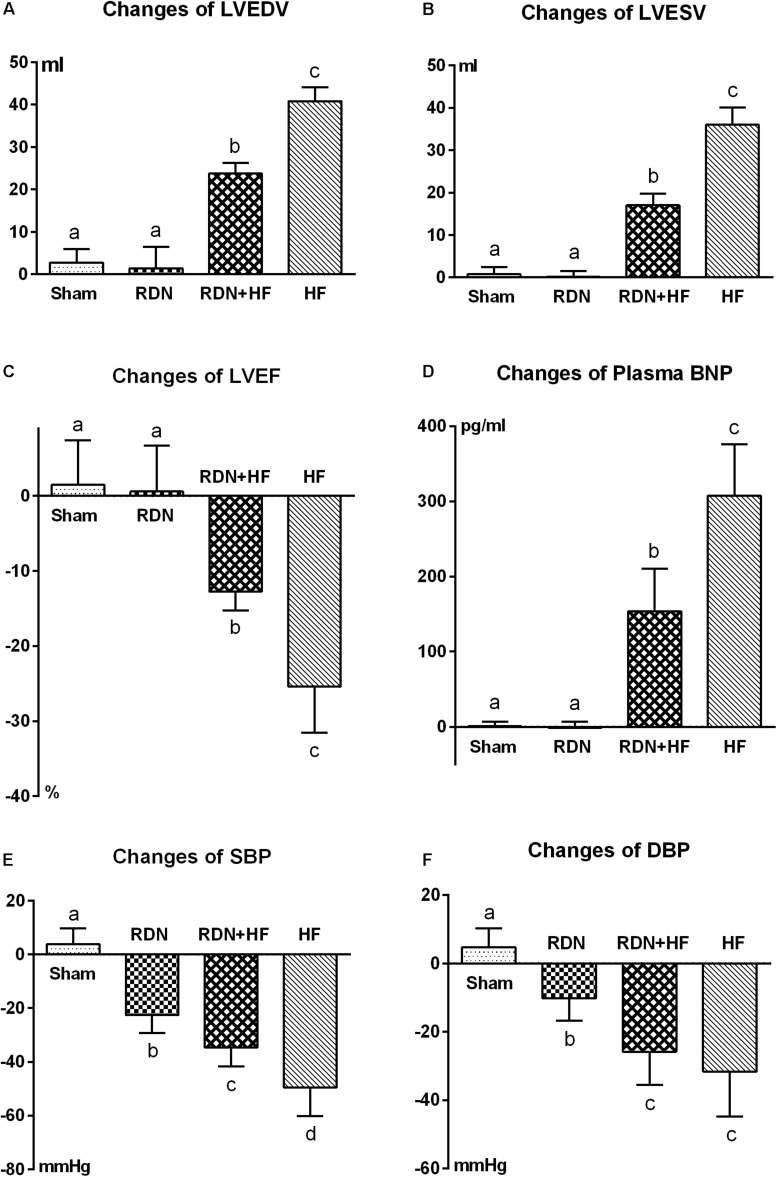
Comparative analyses of echocardiographic measurements **(A–C)**, plasma BNP **(D)**, and BP **(E,F)** among the different study groups after interventions. LVEDV, left ventricular end-diastolic volume; LVESV, left ventricular end-systolic volume; LVEF, left ventricular ejection fraction; BNP, brain natriuretic peptide; BP, blood pressure. The different lowercase letters show significant differences in changes of specific parameter among groups (*p* < 0.05), whereas the same lowercase letters indicate no statistical differences (*p* > 0.05).

However, LVEDV, LVESV, and plasma BNP level in the RDN + HF group and HF-control group were significantly higher than those in the sham-operated group at 4 weeks after interventions (Table 1 and Figure 1), whereas no significant differences were found between the sham-operated group and RDN group (Table 1 and Figure 1). Moreover, the levels of LVEDV, LVESV, and plasma BNP in the HF-control group were markedly higher than those in the RDN + HF group (Table 1 and Figure 1). Additionally, LVEF in the RDN + HF group and HF-control group was significantly lower than that in the sham-operated group at 4 weeks after interventions (Figure 1), with no significant changes observed between the sham-operated group and RDN group (Figure 1). The LVEF in the HF-control group was much lower than that in the RDN + HF group (Figure 1). Therefore, compared with the dogs in the sham-operated group and RDN group, dogs in the RDN + HF group and HF-control group suffered from significant cardiac dysfunction with indicated reduction in LVEF and increase in LVEDV, LVESV, and plasma BNP. The application of the catheter-based RDN technique in the RDN + HF group significantly attenuated the damage caused by continuous right ventricular rapid pacing on cardiac function. However, the application of the RDN technique in the RDN + HF group, compared with dogs in the sham-operated group, could still not absolutely prevent the induction of HF.

As shown in Table 1 and Figure 1, the systolic/diastolic BP in the RDN group, RDN + HF group, and HF-control group was significantly decreased at 4 weeks after interventions, without any significant changes in the sham-operated group. The systolic/diastolic BP in the RDN + HF group and HF-control group was much lower than that in the RDN group (regarding SBP, *p* = 0.039 for RDN + HF vs. RDN, *p* < 0.001 for HF-control vs. RDN; regarding DBP, *p* = 0.026 for RDN + HF vs. RDN, *p* = 0.003 for HF-control vs. RDN; Table 1 and Figure 1). Moreover, the level of systolic BP in the HF-control group was markedly lower than that in the RDN + HF group (*p* = 0.014, Figure 1), whereas no statistical difference was observed in diastolic BP between the RDN + HF group and HF-control group (Figure 1).

Analyses of the circulating levels of AngII, aldosterone, and NE showed no significant differences among the four groups at baseline (Table 1). However, circulating levels of AngII, aldosterone, and NE in the RDN + HF group and HF-control group were significantly increased at 4 weeks after interventions, without any significant change in the sham-operated group (Table 1). Further, no significant differences were found between the sham-operated group and RDN group for the levels of circulating AngII and aldosterone, whereas those of NE were lower in the RDN group compared with the sham-operated group at 4 weeks after interventions (Table 1). Moreover, the circulating levels of AngII, aldosterone, and NE in the HF-control group were strikingly higher than those in the RDN + HF group (Table 1).

### Immunohistochemical Analyses to Determine the Effectiveness of Renal Denervation

The renal artery was stained with hematoxylin–eosin and Masson’s trichrome stains to locate the lesion produced by RDN. In Masson’s trichrome staining, significant hyperplasia of collagen fibers (stained blue) was observed in the ablation area but not in the non-ablation area (Supplementary Figure S1). In hematoxylin–eosin staining of the ablation area, the wavy line of the endothelium was not evident and was also found to be ruptured with apparent medial hyperplasia (Supplementary Figure S1).

**FIGURE 2:**
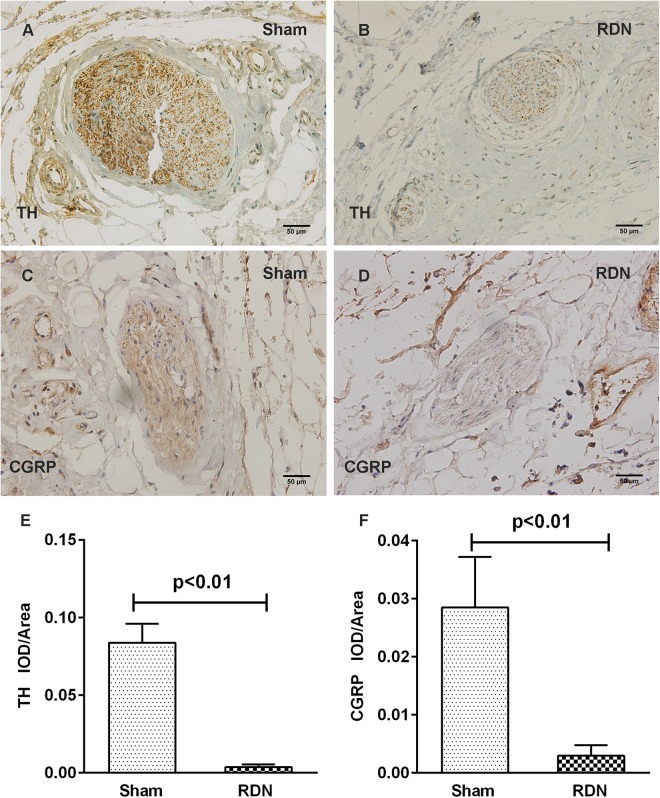
Representative immunohistochemical images and semi-quantitative analysis depicting the impact of catheter-based RDN on the expression of TH and CGRP in renal nerve bundles. RDN, renal denervation; TH, tyrosine hydroxylase, used as a marker for renal efferent sympathetic nerves; CGRP, calcitonin gene-related peptide, used as a marker for renal sensory afferent nerves; IOD, integrated optical density. Immunohistochemical staining for TH and CGRP in renal nerve bundles in the sham-operated group **(A,C)** and RDN group **(B,D)**. The semi-quantitative analysis shows that the average optical density in the RDN group, in comparison with the sham-operated group, for both renal efferent sympathetic nerve **(E)** and renal sensory afferent nerve **(F)** is significantly decreased.

To assess the damage of renal nerves caused by RF energy, immunohistochemical staining was performed using polyclonal antibodies to TH and CGRP. Most of the renal nerve bundles were positive for both renal efferent sympathetic nerves (labeled by TH) and renal sensory afferent nerves (labeled by CGRP) (Supplementary Figure S2). As shown in Figure 2, for the dogs in the sham-operated group without application of RDN, the renal nerve bundles were significantly positive for both renal efferent sympathetic nerves and renal sensory afferent nerves. However, for the renal nerve bundles damaged by RF energy in the RDN group, the average optical density, for both renal efferent sympathetic nerve and renal sensory afferent nerve, was remarkably lower in the RDN group than in the sham-operated group (Figure 2).

Additionally, to systematically evaluate the effectiveness of catheter-based RDN, renal cortical NE concentration was measured using ELISA. The level of renal cortical NE in the RDN group and RDN + HF group was notably lower than that in the sham-operated group (RDN vs. sham-operated, 134.3 ± 37.7 vs. 733.1 ± 183.1 pg/g, *p* = 0.019; RDN + HF vs. sham-operated, 209.9 ± 102.5 vs. 733.1 ± 183.1 pg/g, *p* = 0.018), whereas the renal cortical NE concentration was higher in the HF-control group than in the sham-operated group (1,419.2 ± 248.2 vs. 733.1 ± 183.1 pg/g, *p* = 0.009). However, no significant differences were observed between the RDN and RDN + HF groups (*p* = 0.563).

### Analyses of the Levels of Central Renin–Angiotensin System in the Hypothalamus

**FIGURE 3:**
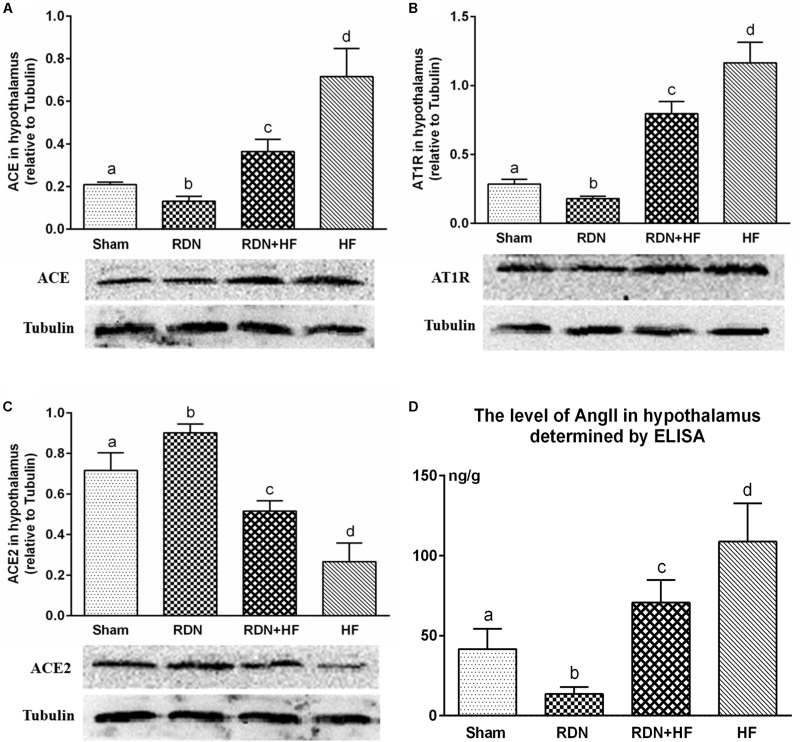
Protein levels of RAS components (ACE/ACE2/AT1R) in the hypothalamus determined by Western blot analysis **(A–C)** and concentration of angiotensin II in the hypothalamus measured *via* ELISA **(D)**. The different lowercase letters show significant differences of the variable among groups (*p* < 0.05), whereas the same lowercase letters show no statistical differences (*p* > 0.05). RAS, renin–angiotensin system; ACE, angiotensin-converting enzyme; AT1R, AngII type 1 receptor.

The level of tissue AngII in the hypothalamus was determined by ELISA. Tissue AngII in the hypothalamus was significantly higher for dogs in the RDN + HF group and HF-control group and lower for dogs in the RDN group compared with the sham-operated group (Figure 3D).

**FIGURE 4:**
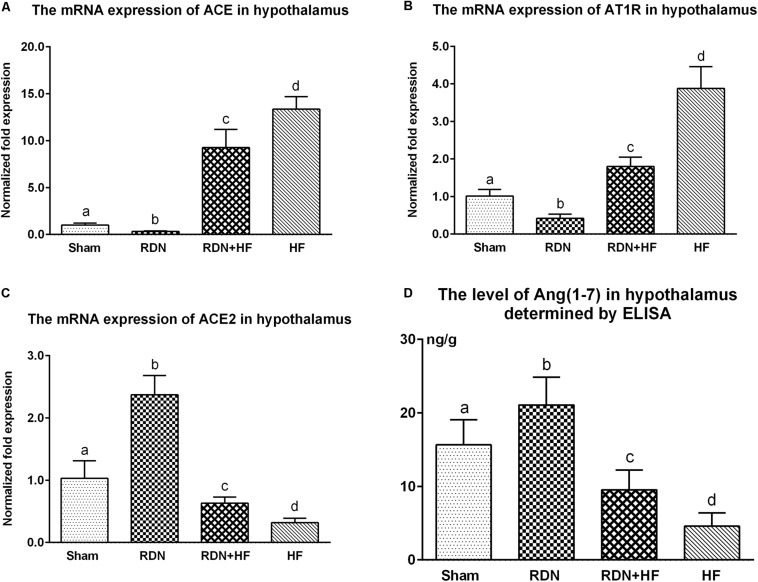
The transcript levels of RAS components (ACE/ACE2/ATIR) in the hypothalamus determined by real-time RT-PCR **(A–C)** and concentration of Ang-(1-7) in the hypothalamus measured *via* ELISA **(D)**. The different lowercase letters show significant differences of the variable among groups (*p* < 0.05), whereas the same lowercase letters show no statistical differences (*p* > 0.05). RAS, renin–angiotensin system; ACE, angiotensin-converting enzyme; AT1R, AngII type 1 receptor; Ang-(1-7), angiotensin-(1-7).

Further, the expression of ACE, AT1R, and ACE2 transcripts and protein was analyzed in the hypothalamus. The protein levels of ACE and AT1R in the hypothalamus of the RDN + HF group and HF-control group were significantly higher than those in the sham-operated group, whereas they were found to be markedly lower in the RDN group than in the sham-operated group (Figure 3). Importantly, the protein levels of ACE and AT1R in the hypothalamus of the RDN + HF group were statistically lower than those of the HF-control group (Figure 3). Additionally, the mRNA expression of ACE and AT1R in the hypothalamus followed the same trend as their protein products (Figure 4).

The mRNA and protein expressions of the RAS component of ACE2 in the hypothalamus were significantly downregulated in the RDN + HF group and HF-control group and upregulated in the RDN group, in comparison with the sham-operated group (Figures 3, 4). The application of RDN in the RDN + HF group, when compared with the HF-control group, significantly attenuated the downregulation of the mRNA and protein levels of ACE2 in the hypothalamus (Figures 3, 4).

The tissue level of Ang-(1-7) in the hypothalamus was also determined by ELISA. The tissue level of Ang-(1-7) in the hypothalamus was significantly lower for dogs in the RDN + HF group and HF-control group and higher for dogs in the RDN group compared with the sham-operated group (Figure 4D).

### Assessment of the Sympathetic Activity of Left Stellate Ganglions

**FIGURE 5:**
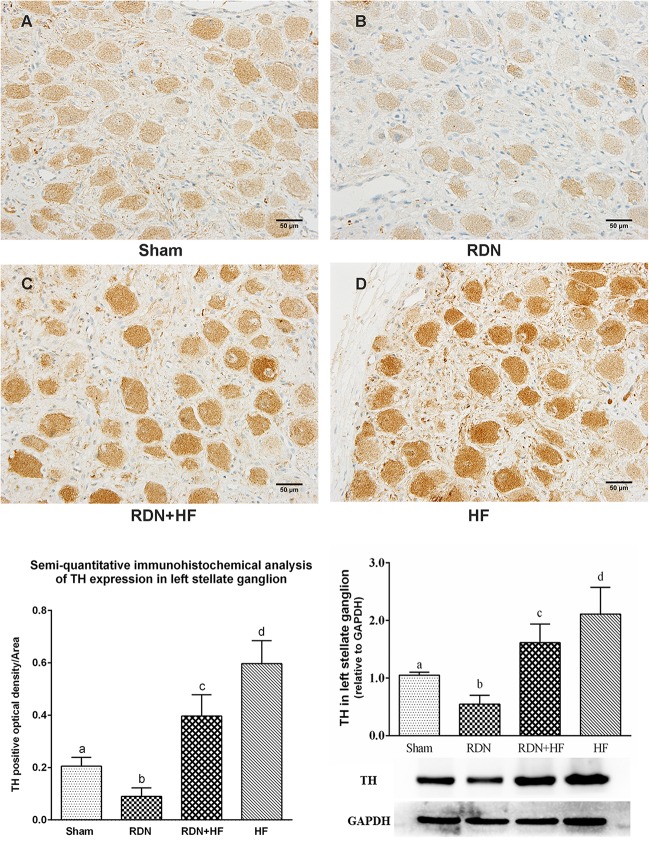
Representative immunohistochemical images **(A–D)** and semi-quantitative and Western blot analysis for the expression of TH in LSG. The different lowercase letters show significant differences of the variable among groups (*p* < 0.05), whereas the same lowercase letters show no statistical differences (*p* > 0.05). TH, tyrosine hydroxylase; LSG, left stellate ganglion.

To investigate the influence of the concerned interventions on the sympathetic activity of LSG, immunohistochemical staining of LSG with polyclonal antibodies to TH was performed. As shown in Figure 5, the IOD of TH in LSG was significantly higher in the RDN + HF group and HF-control group and lower in the RDN group compared with the sham-operated group. Meanwhile, the expression of TH in LSG in the RDN + HF group, compared with the HF-control group, was notably attenuated by the application of the catheter-based RDN technique (Figure 5). Importantly, the protein expression level of TH in LSG corroborates the aforementioned immunohistochemical analysis results (Figure 5).

### Effects of Renal Denervation on Cardiac Sympathetic Nerve Remodeling

**FIGURE 6:**
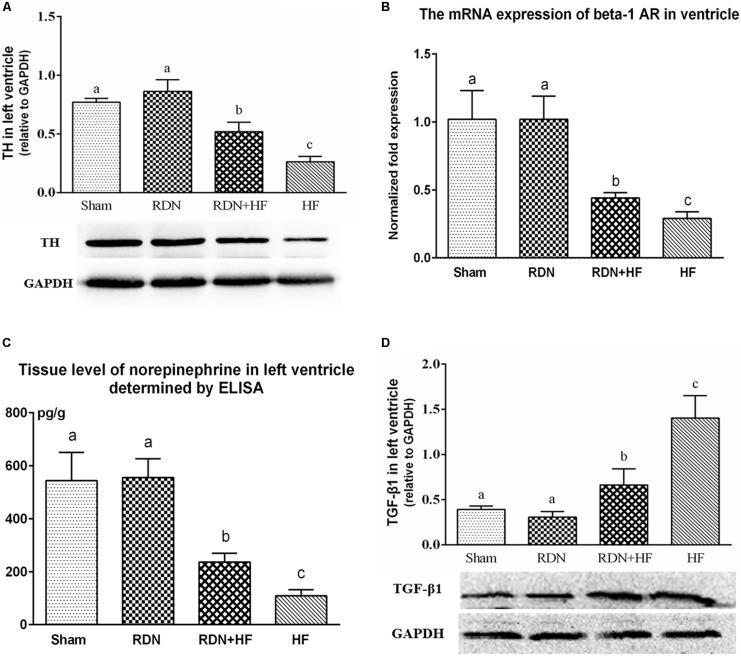
Protein levels of TH **(A)** and TGF-β1 **(D)** in the left ventricle determined by Western blot analysis and mRNA expressions of ventricular β1-AR **(B)** detected by real-time RT-PCR and tissue level of norepinephrine in left ventricle determined by ELISA **(C)**. The different lowercase letters show significant differences of the variable among groups (*p* < 0.05), whereas the same lowercase letters show no statistical differences (*p* > 0.05). TH, tyrosine hydroxylase; TGF-β1, transforming growth factor-β1; β1-AR, beta-1-adrenergic receptor.

The protein levels of TH in the left ventricle of the RDN + HF and HF-control groups were significantly lower than those in the sham-operated group, with no significant differences between the RDN group and sham-operated group (Figure 6A). Further, the protein expression level of TH in the left ventricle of the HF-control group was much lower than that of the RDN + HF group (Figure 6A).

Moreover, quantitative RT-PCR analyses showed that the gene levels of the β1-AR in left ventricle, normalized to mRNA expression of GAPDH, were lower in the RDN + HF group and HF-control group, compared with the sham-operated group (Figure 6B). However, no notable difference was found between the RDN group and sham-operated group (Figure 6B). The gene level of β1-AR in the left ventricle of the HF-control group was found to be markedly lower than that of the RDN + HF group (Figure 6B).

Additionally, the tissue level of NE in the left ventricle was also determined using ELISA. It was observed that the tissue level of NE in the left ventricle of the RDN + HF group and HF-control group was distinctly lower than that of the sham-operated group (Figure 6C). However, no significant differences were observed between the RDN group and sham-operated group (Figure 6C). Meanwhile, the tissue level of NE in the left ventricle of the HF-control group was found to be significantly lower than that of the RDN + HF group (Figure 6C).

### Effects of Renal Denervation on Cardiac Fibrosis

**FIGURE 7:**
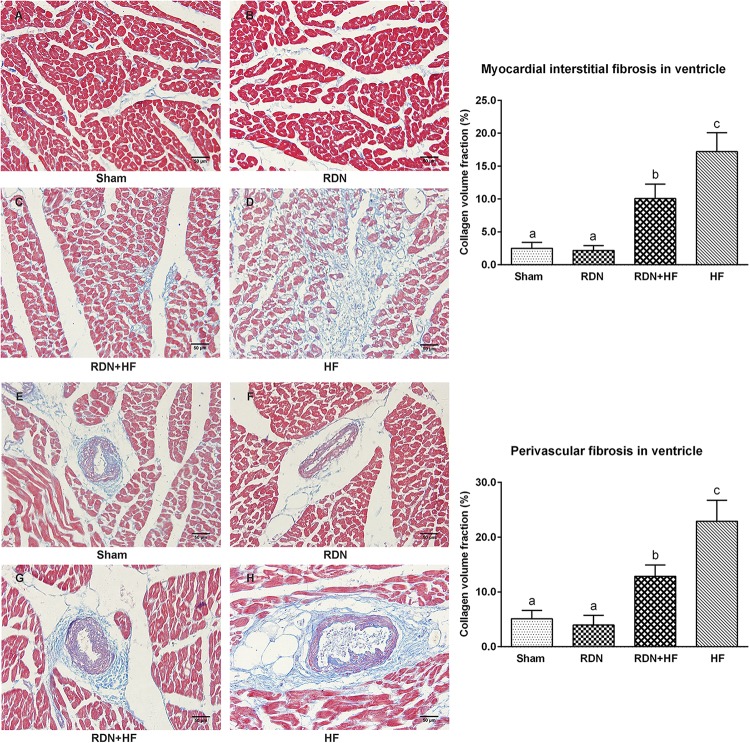
Representative Masson’s staining images and semi-quantitative analysis for the evaluation of cardiac fibrosis. Masson’s staining was performed in both interstitial cardiac tissue **(A–D)** and the perivascular cardiac tissue **(E–H)**. The fibrosis area is stained blue. The different lowercase letters show significant differences of collagen volume fraction among groups (*p* < 0.05), whereas the same lowercase letters show no statistical differences (*p* > 0.05).

Masson’s trichrome staining was performed to assess left ventricular fibrosis. It could be observed that interstitial cardiac fibrosis was markedly significant in the RDN + HF group and HF-control group, compared with the sham-operated group (Figure 7). However, the interstitial cardiac fibrosis in the HF-control group was distinctly evident compared with that in the RDN + HF group (Figure 7). No significant differences were observed between the RDN group and sham-operated group (Figure 7). Further, perivascular cardiac fibrosis was found to be consistent with the interstitial cardiac fibrosis results (Figure 7).

As a key mediator in stimulating the activation of cardiac fibroblasts and cardiac fibrosis, TGF-β1 level in the left ventricular tissues was determined by WB. The protein levels of TGF-β1 in the RDN + HF and HF-control groups were significantly higher than those in the sham-operated group, whereas no notable differences were found between the RDN group and sham-operated group (Figure 6D). Additionally, the TGF-β1 protein level in the HF-control group was markedly higher than that in the RDN + HF group (Figure 6D).

## Discussion

The major findings of the current study are summarized as follows: (1) catheter-based RDN could simultaneously damage the renal efferent sympathetic nerves and afferent sensory nerves. (2) Application of the RDN technique significantly attenuated the upregulation of the AngII/ACE/AT1R axis and downregulation of Ang-(1-7)/ACE2 in the hypothalamus in the RDN + HF group than in the HF-control group. (3) Application of the RDN technique suppressed the progression of cardiac fibrosis and sympathetic nerve remodeling, which was evidenced by attenuating the reduction of ventricular expression of β1-AR and TGF-β1. These parameters contribute to improving the cardiac dysfunction induced by right ventricular rapid pacing.

This study also shows that both efferent sympathetic nerve fibers and afferent sensory nerve fibers are mostly contained in the same nerve bundles (van Amsterdam et al., 2016). Therefore, the catheter-based RDN technique damages both the renal efferent sympathetic nerves and also the renal afferent sensory nerves *via* the delivery of RF energy.

Extensive pieces of evidence have demonstrated that central RAS plays a critical role in regulating the central sympathetic outflow (Fujisawa et al., 2011; Zucker et al., 2014). Reports indicate that central AngII, *via* activation of local AT1Rs, significantly augments the central sympathetic outflow, which can be effectively suppressed by AT1R antagonists (Yoshimura et al., 2000; Campese et al., 2002; Gao et al., 2004; Fujisawa et al., 2011; Zucker et al., 2014). Meanwhile, the balance between central ACE and ACE2 in the brain also has a major influence on the central regulation of sympathetic outflow. It is known that the increase of central ACE induces sympathetic outflow by promoting the generation of AngII, whereas overexpression of ACE2 results in the decrease of sympathetic outflow by compromising the level of AngII and downregulating the expression of central AT1Rs (McKinley et al., 2003; Francis et al., 2004; Kar et al., 2010; Xiao et al., 2011; Zucker et al., 2014).

In the present study, using the plasma NE concentrations and protein levels of TH in LSG as indirect indices for evaluating the central sympathetic outflow to the peripheral circulation system and heart, we showed that the expression level of TH in LSG and the plasma NE concentrations were significantly increased in the RDN + HF and HF-control groups. However, the application of the catheter-based RDN technique in the RDN + HF group significantly attenuated the increased expression of TH in LSG and the plasma NE concentrations. The blood sample for determining plasma NE concentration was collected from anesthetized animals in the present study, which may have an influence on their plasma concentrations. However, the anesthesia protocol among the dogs enrolled in this study is similar, thereby making the results of blood test among the groups permissible to comparison. Meanwhile, previous published studies have shown that RDN can decrease the plasma NE concentrations (Schlaich et al., 2009; Lu et al., 2015; Chen et al., 2016b), MSNA (Hering et al., 2013), and cardiac sympathetic activity (Donazzan et al., 2016). These observations, which indirectly indicate that the catheter-based RDN technique effectively decreases the sympathetic outflow from brain to the heart and peripheral circulation system, are consistent with our current findings. Furthermore, it has been demonstrated that renal afferent sensory nerves are mainly projected to the hypothalamus areas (Calaresu and Ciriello, 1981; Xu et al., 2012, 2015). In this line, the present study shows that the RDN technique significantly attenuates the overactivity of central RAS in the hypothalamus area, manifested as reduced central AngII level, downregulated transcript and protein levels of ACE and AT1R, and simultaneous increase in the mRNA and protein expressions of ACE2. Thus, simultaneously considering the previous discussion for the role of central RAS in regulating the central sympathetic outflow, the results of our current study indirectly imply that, through concomitant damage of the renal afferent nerves, the catheter-based RDN technique decreases the central sympathetic outflow to the heart and peripheral circulation system by attenuating the overactivity of central RAS in the hypothalamus.

Interestingly, the research conducted by Fujisawa et al. (2011) showed that excessive activation of the renal afferent nerves induced significant elevation of central sympathetic outflow and resulted in the corresponding increase in BP and renal sympathetic nerve activity. However, intracerebroventricular injection of AT1R antagonists significantly decreased the sympathetic outflow and suppressed the vasopressor and sympathoexcitatory responses to excessive activation of renal afferent nerves (Fujisawa et al., 2011). Furthermore, similar to the intracerebroventricular injection of AT1R antagonists, surgical RDN can also suppress the sympathoexcitatory effects induced by excessive renal afferent nerve activation (Fujisawa et al., 2011). Therefore, the research conducted by Fujisawa et al. (2011) also indicated that renal afferent nerves contributed to the regulation of sympathetic outflow through central RAS-dependent pathways. Meanwhile, by damaging the renal afferent nerves, the RDN technique attenuates the central sympathetic outflow to the heart and the peripheral circulation system (Fujisawa et al., 2011).

Previous studies have demonstrated that both renal efferent sympathetic nerves and renal afferent sensory nerves are excessively activated in HF, which also promotes the progression of HF, thereby generating a vicious circle (Booth et al., 2015; Schiller et al., 2015). Thus, renal nerves, including efferent sympathetic nerves and the afferent sensory nerves, should be considered as important therapeutic targets for HF. As shown in our present study, the application of the RDN technique significantly suppresses the cardiac fibrosis and sympathetic nerve remodeling procedure, finally attenuating the cardiac dysfunction induced by right ventricular rapid pacing.

To interpret the results of the present study, some limitations must be considered. First, this study mainly focused on the role of the central RAS in the hypothalamus in the regulation of sympathetic outflow. However, published research indicates that surgical RDN can also regulate the central sympathetic outflow through the influence on central nitric oxide synthase (Patel et al., 2016; Zheng et al., 2018). Thus, the RDN technique attenuates the central sympathetic outflow partly through the central RAS-dependent pathway. Meanwhile, the molecular biological analysis of central RAS in the hypothalamus was performed using the whole hypothalamus, as the lack of stereotaxic apparatus for brain research in dogs restricted the isolation of the specific autonomic nuclei from the hypothalamus. Additionally, the evaluation of sympathetic outflow in our manuscript was indirectly indicated by the level of plasma NE concentrations and the sympathetic nerve activity in LSG, rather than directly recording the activity of peripheral sympathetic fibers. Finally, the present study was based on animal research. Although this study has provided additional evidences for the application of the catheter-based RDN technique in the treatment of HF, the long-term beneficial effects of RDN on HF still can only be confirmed by large-scale, multicenter, double-blinded, randomized, controlled clinical trials.

## Conclusion

In conclusion, along with the damage to renal efferent sympathetic nerves, the catheter-based RDN technique can also simultaneously disrupt the renal afferent sensory nerves, thereby attenuating the overactivity of central RAS in the hypothalamus, decreasing the sympathetic activity of LSG and ventricle, suppressing the progression of cardiac fibrosis and sympathetic nerve remodeling, and eventually contributing to the improvement of the cardiac dysfunction induced by right ventricular rapid pacing in canines.

## Data Availability Statement

All datasets generated for this study are included in the article/Supplementary Material.

## Ethics Statement

The animal study was reviewed and approved by the Animal Research Ethics Committee of Chongqing Medical University.

## Author Contributions

W-JC, HL, Z-HW, CL, J-QF, Z-LW, Y-PX, BZ, LG, QL, Z-YL, and Y-HY carried out the study design, performed the manuscript writing and revising, and approved the final manuscript. W-JC, HL, Z-HW, CL, and Z-YL carried out the operation and data collection. W-JC, HL, J-QF, Z-LW, Y-PX, LG, QL, Z-YL, and Y-HY performed the data analysis. All authors have read and approved the final manuscript.

## Conflict of Interest

The authors declare that the research was conducted in the absence of any commercial or financial relationships that could be construed as a potential conflict of interest.

## References

[B1] Böhm M. Ewen S. Kindermann I. Linz D. Ukena C. Mahfoud F. (2014). Renal denervation and heart failure. *Eur. J. Heart Fail.* 16 608–613. 10.1002/ejhf.83 24644008

[B2] Booth L. C. May C. N. Yao S. T. (2015). The role of the renal afferent and efferent nerve fibers in heart failure. *Front. Physiol.* 6:270. 10.3389/fphys.2015.00270 26483699 PMC4589650

[B3] Calaresu F. R. Ciriello J. (1981). Renal afferent nerves affect discharge rate of medullary and hypothalamic single units in the cat. *J. Auton. Nerv. Syst.* 3 311–320. 10.1016/0165-1838(81)90072-2 7276438

[B4] Campese V. M. Ye S. Zhong H. (2002). Downregulation of neuronal nitric oxide synthase and interleukin-1beta mediates angiotensin II-dependent stimulation of sympathetic nerve activity. *Hypertension* 39 519–524. 10.1161/hy0202.102815 11882601

[B5] Chen W. Du H. Lu J. Ling Z. Long Y. Xu Y., et al. (2016a). Renal artery vasodilation may be an indicator of successful sympathetic nerve damage during renal denervation procedure. *Sci. Rep.* 6:37218. 10.1038/srep37218 27849014 PMC5110962

[B6] Chen W. Ling Z. Xu Y. Liu Z. Su L. Du H. (2016b). Preliminary effects of renal denervation with saline irrigated catheter on cardiac systolic function in patients with heart failure: a prospective, randomized, controlled, pilot study. *Catheter. Cardiovasc. Interv.* 89 E153–E161. 10.1002/ccd.26475 27143319

[B7] Clayton S. C. Haack K. K. Zucker I. H. (2011). Renal denervation modulates angiotensin receptor expression in the renal cortex of rabbits with chronic heart failure. *Am. J. Physiol. Renal Physiol.* 300 F31–F39. 10.1152/ajprenal.00088.2010 20962112 PMC3023215

[B8] Davies J. E. Manisty C. H. Petraco R. Barron A. J. Unsworth B. Mayet J. (2013). First-in-man safety evaluation of renal denervation for chronic systolic heart failure: primary outcome from REACH-Pilot study. *Int. J. Cardiol.* 162 189–192. 10.1016/j.ijcard.2012.09.019 23031283

[B9] DiBona G. F. Sawin L. L. (1991). Role of renal nerves in sodium retention of cirrhosis and congestive heart failure. *Am. J. Physiol.* 260 R298–R305. 1996717 10.1152/ajpregu.1991.260.2.R298

[B10] Donazzan L. Mahfoud F. Ewen S. Ukena C. Cremers B. Kirsch C. M. (2016). Effects of catheter-based renal denervation on cardiac sympathetic activity and innervation in patients with resistant hypertension. *Clin. Res. Cardiol.* 105 364–371. 10.1007/s00392-015-0930-4 26493305

[B11] Francis J. Wei S. G. Weiss R. M. Felder R. B. (2004). Brain angiotensin-converting enzyme activity and autonomic regulation in heart failure. *Am. J. Physiol. Heart Circ. Physiol.* 287 H2138–H2146. 10.1152/ajpheart.00112.2004 15475532

[B12] Fujisawa Y. Nagai Y. Lei B. Nakano D. Fukui T. Hitomi H. (2011). Roles of central renin-angiotensin system and afferent renal nerve in the control of systemic hemodynamics in rats. *Hypertens. Res.* 34 1228–1232. 10.1038/hr.2011.115 21796126

[B13] Gao L. Wang W. Li Y. L. Schultz H. D. Liu D. Cornish K. G. (2004). Superoxide mediates sympathoexcitation in heart failure: roles of angiotensin II and NAD(P)H oxidase. *Circ. Res.* 95 937–944. 10.1161/01.RES.0000146676.04359.64 15459075

[B14] Hering D. Lambert E. A. Marusic P. Walton A. S. Krum H. Lambert G. W. (2013). Substantial reduction in single sympathetic nerve firing after renal denervation in patients with resistant hypertension. *Hypertension* 61 457–464. 10.1161/HYPERTENSIONAHA.111.00194 23172929

[B15] Hopper I. Gronda E. Hoppe U. C. Rundqvist B. Marwick T. H. Shetty S. (2017). Sympathetic response and outcomes following renal denervation in patients with chronic heart failure: 12-month outcomes from the symplicity HF feasibility study. *J. Card. Fail.* 23 702–707. 10.1016/j.cardfail.2017.06.004 28645757

[B16] Kar S. Gao L. Zucker I. H. (2010). Exercise training normalizes ACE and ACE2 in the brain of rabbits with pacing-induced heart failure. *J. Appl. Physiol.* 108 923–932. 10.1152/japplphysiol.00840.2009 20093667 PMC2853198

[B17] Lu J. Wang Z. Zhou T. Chen S. Chen W. Du H. (2015). Selective proximal renal denervation guided by autonomic responses evoked via high-frequency stimulation in a preclinical canine model. *Circ. Cardiovasc. Interv.* 8:e001847. 10.1161/CIRCINTERVENTIONS.115.001847 26058393

[B18] Mahfoud F. Lüscher T. F. Andersson B. Baumgartner I. Cifkova R. Dimario C. (2013). Expert consensus document from the European society of cardiology on catheter-based renal denervation. *Eur. Heart J.* 34 2149–2157. 10.1093/eurheartj/eht154 23620497

[B19] McKinley M. J. Albiston A. L. Allen A. M. Mathai M. L. May C. N. McAllen R. M. (2003). The brain renin-angiotensin system: location and physiological roles. *Int. J. Biochem. Cell Biol.* 35 901–918. 10.1016/s1357-2725(02)00306-0 12676175

[B20] Patel K. P. Xu B. Liu X. Sharma N. M. Zheng H. (2016). Renal denervation improves exaggerated sympathoexcitation in rats with heart failure: a role for neuronal nitric oxide synthase in the paraventricular nucleus. *Hypertension* 68 175–184. 10.1161/HYPERTENSIONAHA.115.06794 27185748 PMC4900899

[B21] Polhemus D. J. Trivedi R. K. Gao J. Li Z. Scarborough A. L. Goodchild T. T. (2017). Renal sympathetic denervation protects the failing heart via inhibition of neprilysin activity in the kidney. *J. Am. Coll. Cardiol.* 70 2139–2153. 10.1016/j.jacc.2017.08.056 29050562

[B22] Schiller A. M. Pellegrino P. R. Zucker I. H. (2015). The renal nerves in chronic heart failure: efferent and afferent mechanisms. *Front. Physiol.* 6:224. 10.3389/fphys.2015.00224 26300788 PMC4528173

[B23] Schlaich M. P. Hering D. Sobotka P. A. Krum H. Esler M. D. (2012). Renal denervation in human hypertension: mechanisms, current findings, and future prospects. *Curr. Hypertens. Rep.* 14 247–253. 10.1007/s11906-012-0264-9 22457244

[B24] Schlaich M. P. Sobotka P. A. Krum H. Lambert E. Esler M. D. (2009). Renal sympathetic-nerve ablation for uncontrolled hypertension. *N. Engl. J. Med.* 361 932–934. 10.1056/NEJMc0904179 19710497

[B25] Van Amsterdam W. A. Blankestijn P. J. Goldschmeding R. Bleys R. L. (2016). The morphological substrate for renal denervation: nerve distribution patterns and parasympathetic nerves. a post-mortem histological study. *Ann. Anat.* 204 71–79. 10.1016/j.aanat.2015.11.004 26617159

[B26] Villarreal D. Freeman R. H. Johnson R. A. Simmons J. C. (1994). Effects of renal denervation on postprandial sodium excretion in experimental heart failure. *Am. J. Physiol.* 266 R1599–R1604. 8203638 10.1152/ajpregu.1994.266.5.R1599

[B27] Xiao L. Gao L. Lazartigues E. Zucker I. H. (2011). Brain-selective overexpression of angiotensin-converting enzyme 2 attenuates sympathetic nerve activity and enhances baroreflex function in chronic heart failure. *Hypertension* 58 1057–1065. 10.1161/HYPERTENSIONAHA.111.176636 22025374 PMC3224814

[B28] Xu B. Zheng H. Liu X. Patel K. P. (2015). Activation of afferent renal nerves modulates RVLM-projecting PVN neurons. *Am. J. Physiol. Heart Circ. Physiol.* 308 H1103–H1111. 10.1152/ajpheart.00862.2014 25637549 PMC4551125

[B29] Xu B. Zheng H. Patel K. P. (2012). Enhanced activation of RVLM-projecting PVN neurons in rats with chronic heart failure. *Am. J. Physiol. Heart Circ. Physiol.* 302 H1700–H1711. 10.1152/ajpheart.00722.2011 22307669 PMC3330797

[B30] Yoshimura R. Sato T. Kawada T. Shishido T. Inagaki M. Miyano H. (2000). Increased brain angiotensin receptor in rats with chronic high-output heart failure. *J. Card. Fail.* 6 66–72. 10.1016/s1071-9164(00)00013-0 10746821

[B31] Zheng H. Katsurada K. Liu X. Knuepfer M. M. Patel K. P. (2018). Specific afferent renal denervation prevents reduction in neuronal nitric oxide synthase within the paraventricular nucleus in rats with chronic heart failure. *Hypertension* 72 667–675. 10.1161/HYPERTENSIONAHA.118.11071 30012866 PMC6202134

[B32] Zucker I. H. Xiao L. Haack K. K. (2014). The central renin-angiotensin system and sympathetic nerve activity in chronic heart failure. *Clin. Sci.* 126 695–706. 10.1042/CS20130294 24490814 PMC4053944

