# Supplementary material for: The Impact of Renal Denervation on the Progression of Heart Failure in a Canine Model Induced by Right Ventricular Rapid Pacing
Source: Front Physiol. 2020 Jan 31;10:1625. doi: 10.3389/fphys.2019.01625 (PMC7004968; doi:10.3389/fphys.2019.01625)
Supplement: Supplementary file 1 [file Data_Sheet_1.pdf]

Supplementary Material

1. Detailed Materials and Methods

1.1 Experimental Animals and Study Protocol

The Chinese Kunming dogs which were obtained from Chengdu Chinese Kunming Dogs Breeding Base in Sichuan province in China, were used as experimental animals in this study, and standardly fed in Laboratory Animal Center of Chongqing Medical University. Written informed consent was obtained from Chengdu Chinese Kunming Dogs Breeding Base for the participation of these dogs in this animal study. Reasons of using Chinese Kunming dogs as experimental animals[1-3], the source, breeding, feeding conditions and the experimental protocol were reviewed and approved by the animal research ethics committee of Chongqing Medical University in accordance with the guidelines of the National Institutes of Health and of the Declaration of Helsinki for the Care and Use of Laboratory Animals.

All enrolled Chinese Kunming dogs aged 3-4 years, weighed 25-35 kg. A total of 24 Chinese Kunming dogs were enrolled and randomly assigned into 4 groups: Sham-operated group (n=6), RDN group (n=6), RDN+HF group (n=6), and HF-control group (n=6). The dogs in RDN group received renal artery angiography and catheter-based RDN with the use of a 6F renal artery dedicated open-irrigated ablation catheter (AquaSense, Synaptic Medical Limited, Beijing, China). The dogs in the Sham-operated group were given the renal artery angiography followed by the placement of ablation catheter without radiofrequency (RF) energy delivery. For the dogs in the RDN+HF group, a high-rate cardiac pacemaker (Fudan University, Shanghai, China) attaching a ventricular endocardial

pacing electrode (St. Jude Medical, Inc.) was implanted with continuous right ventricular pacing at 250 beats/min plus the renal artery angiography and catheter-based RDN procedures. The dogs in the HF-control group simultaneously received continuous right ventricular pacing at 250 beats/min as well as the renal artery angiography and placement of ablation catheter without energy delivery.

Anesthesia was induced with 3% sodium pentobarbital (30 mg/kg) intraperitoneally and maintained by continuously venous injection at a dosage of 5 mg/kg/h via trace syringe pump. To prevent perioperative infections, one million units of penicillin G were given intramuscularly 30 minutes prior to surgery and immediately after the surgery, followed by every 12 hours for three days. Regarding the post-operation analgesia therapy, carprofen was orally given at the dose of 4 mg/kg for 3 days to relieve perioperative pain. All enrolled dogs received transthoracic echocardiographic examinations to assess the cardiac structure and function at baseline. Bilateral femoral arteries were punctured under sterile conditions, and 2000 IU unfractionated heparin was administered via right femoral artery. The surface electrocardiogram (ECG) and invasive left femoral artery pressure were continuously monitored throughout the interventional procedure using a Multichannel Electrophysiology Management System (Sichuan Jinjiang Electronic Science and Technology Corporation, Chengdu, China). Meanwhile, blood samples (10 ml) were also collected from femoral vein for the purpose of measuring the plasma brain natriuretic peptide (BNP), the norepinephrine (NE) concentrations, and the circulating levels of ANG II and aldosterone.

After interventional procedures, all dogs were fed in Chongqing Medical University Laboratory Animal Center with standard food and water. Their conditions were observed daily. Dogs in RDN+HF group and HF-control group received weekly surface ECG to verify the continuous 1:1 ventricular capture. After 4 weeks' standard feeding, all dogs were anesthetized to follow up the transthoracic echocardiographic examinations, the surface ECG, the invasive femoral artery pressure,

the renal artery angiography, the levels of plasma BNP and NE concentrations, and the circulating levels of angiotensin II and aldosterone.

At the end point of the experiment, dogs were euthanized with an over-dose of sodium pentobarbital (200 mg/kg). The renal artery, kidney, brain tissue, left stellate ganglion (LSG), and heart were harvested immediately. Renal artery and part of LSG were fixed in 10% formalin solution for histopathological and immunohistochemical analyses. Kidney and brain tissue as well as ventricular tissue and LSG were immediately frozen in liquid nitrogen for molecular biological analyses. The interventricular septum cut from the heart was fixed in 10% formalin solution for the Masson's trichrome stains.

1.2 Renal Artery Angiography and Renal Denervation

Bilateral renal artery angiography was performed via right femoral artery to all dogs. After eliminating the renal artery abnormalities (such as severe distortion or stenosis (diameter < 4mm)), the 6F ablation catheter was positioned into the renal artery via right femoral access. Catheter-based RDN was performed from distal to proximal lumen of the renal artery trunk by point to point burns both longitudinally and rotationally. Eight to twelve lesions were created in each renal artery as per its length. Impedance, power and temperature were continuously monitored. The temperature was set to 45 °C, with 10 watts of RF energy and 70 seconds' duration for each lesion. Saline was irrigated at 3 ml/min to cool down the temperature of tissue-electrode interface during RF energy delivery using the Vation-Cool Pump (Sichuan Jinjiang Electronic Science and Technology Corporation, Chengdu, China).

1.3 Induction of Heart Failure

In RDN+HF group and HF-control group, HF was induced simultaneously by continuous right ventricular rapid pacing. Under fluoroscopy, the endocardial pacing electrode was inserted into right ventricle through jugular vein and positioned in ventricular apex. After verification of successful ventricular capture, proximal terminal of the electrode was attached to the high-rate cardiac pacemaker. Both the pacemaker and attached electrode were implanted and fixed in subcutaneous pocket in the neck. All dogs in RDN+HF group and HF-control group were paced at 250 beats/min for 4 weeks to induce HF. The surface ECG was performed every week to ensure the continuous 1:1 ventricular capture. Transthoracic echocardiographic examination and the measurement of BNP were performed both at baseline and 4 weeks after continuous rapid pacing to evaluate the cardiac function.

1.4 Transthoracic Echocardiographic Examination

Transthoracic echocardiographic examination was performed on all enrolled dogs by using iE33 ultrasound systems (Philips, Netherlands) at baseline and 4 weeks after interventions. Left ventricular end-diastolic volume (LVEDV), left ventricular end-systolic volume (LVESV), and left ventricular ejection fraction (LVEF) were measured by an echocardiography expert who was kept from the dogs' grouping. Simpson's method was used to calculate the LVEF.

1.5 Histopathology and Immunohistochemistry

Harvested from the dogs, the bilateral renal arteries with surrounding tissues, part of LSG, and interventricular septum were fixed in 10% formalin solution for 24-48h, subjected to alcohol dehydration, embedded in paraffin, and then sectioned.

Renal arteries were sectioned at approximate 5-mm intervals from distal (kidney) to proximal (aorta) and cut into 5-μm slices. A total of 10 consecutive slices were obtained from each section. Slices from each section were stained with hematoxylin-eosin and Masson's trichrome stains to locate the lesion produced by RDN, which were also stained with polyclonal antibodies to tyrosine hydroxylase (TH, AB117112, Abcam, used at 1:1000) and calcitonin gene related peptide (CGRP, NO.250602, Abbiotec, used at 1:100) to assess the damage induced by RDN on the renal efferent sympathetic and afferent sensory nerves. The immunoreactivity and protein expression of TH or CGRP in renal nerve bundles were analyzed and quantified on the basis of the integrated optical density (IOD) of the TH|CGRP-positive nerves using computerized image analysis system (Image-Pro Plus 6.0, Media Cybernetics, Inc., USA). Data are presented as mean IOD of TH|CGRP-positive nerves per unit area of nerve bundle, while the area of nerve bundle was also measured by Image-Pro Plus and expressed as pixels.

Each LSG tissue was sectioned into 5 μm slices at 2- to 3-mm intervals. Slices were stained with TH (AB117112, Abcam, used at 1:1000) to investigate the influence of RDN on sympathetic outflow from brain to heart. The immunoreactivity and protein expression of TH in LSG were also analyzed and quantified on the basis of IOD of the TH-positive nerves using Image-Pro Plus software. At least five slices and five microscopic fields of each slice were randomly selected under x400 magnification. Data were presented as mean IOD of TH-positive nerves/Field.

Each interventricular septum tissue was sectioned into 5 μm slices at 3- to 4-mm intervals. Slices were stained with Masson's trichrome to assess the influence of RDN on cardiac fibrosis. Collagen volume fraction, which was expressed as the percentage of fibrosis area per microscopic field with the use of the Image-Pro Plus 6.0, was used to evaluate the extents of cardiac fibrosis. Five

slices and at least five microscopic fields per slice were randomly selected to perform the above analyses of collagen volume fraction.

1.6 Western Blot Analysis

The expression levels of ACE, ACE2, and AT1R protein in hypothalamus, TH protein in LSG, and the levels of TH protein and TGF-β in ventricle were assayed by Western blot (WB). The brain tissue, the LSG and ventricle were unfrozen on the ice before performing immunoblotting. The hypothalamus, weighting 1000 mg and locating at posterior of the optic chiasm and dorsal of the hypophysis, was cut and obtained from the brain according to the anatomy of dogs. The 200 mg of hypothalamus, LSG and ventricular tissues were respectively sectioned and used for immunoblotting.

As previously described, tissues from hypothalamus, LSG, or ventricle were homogenized in lysis buffer (P0013, Beyotime Biotechnology, China) containing a 1:100 dilution of protease inhibitor, and were purified by centrifugation (12,000g for 15 min at 4 °C). Protein concentrations were detected by the BCA Protein Assay Kit (P0012, Beyotime Biotechnology, China). Equivalent amount of protein was separated by electrophoresis, and electro-transferred to PVDF membranes. After being blocked by 5% milk in Tris-buffered saline with Tween 20 (TBST), the membranes were incubated overnight at 4 °C with primary antibody including anti-ACE (AB11737, Abcam, used at 1:50), anti-ACE2 (SAB2100025, Sigma, used at 1:100), anti-AT1R (SAB2100073, Sigma, used at 1:200), and anti-TH (AB117112, Abcam, used at 1:500). Anti-tubulin (NO.10068-1-AP, Proteintech, used at 1:1000) and Anti-GAPDH (NO.10068-1-AP, Proteintech, used at 1:2000) were used as an internal control. After incubated overnight with primary antibody and washed by TBST, the membranes were incubated with horseradish peroxidase-conjugated secondary antibodies for 2 hours at room temperature. Using an enhanced chemiluminescence method, the protein bands on the

membranes were detected and analyzed by the Bio-Rad Gel Imaging System (Bio-Rad, CA, USA) and Quantity One software (Bio-Rad, CA, USA) respectively. The expression levels were quantified by Quantity-One software with normalized to the expression of tubulin or GAPDH as internal control.

1.7 RNA Isolation and Real-Time Reverse Transcription Polymerase Chain Reaction (RT-PCR)

Total RNA was extracted from hypothalamus tissue and ventricular tissue using the RNAiso Plus (No.9109, Takara, Dalian, China) in accordance with the instruction manual and quantified using NanoDrop 2000 spectrophotometer (Thermo Scientific, USA). Complementary DNA was generated using the PrimeScript™ RT reagent Kit (NO.RR047A, Takara, Dalian, China). Real-time RT-PCR was performed on complementary DNA using the SYBR® Premix Ex Taq™ II Kit (NO. RR820A, Takara, Dalian, China) in a C1000 Touch™ Thermal Cycler (Bio-Rad, USA). The mixtures were heated at 95 °C for 30 seconds, followed by 40 cycles at 95 °C for 5 seconds and 60 °C for 30 seconds, and then the melt curve was inserted. All reactions were performed in triplicate, taking GAPDH as an internal control. Data were quantified by Ct values, where Ct is defined as the threshold cycle of PCR at which amplified product was first detected and expressed as ratio of target to control. The primer sequences of all genes are presented in **Supplemental Table 1.** The relative expression ratio of target genes was calculated using comparative Ct method.

1.8 Enzyme Linked ImmunoSorbent Assay (ELISA)

After centrifugation at 3000g for 15 min at 4 °C, the plasma was obtained and stored in the fridge at -80 °C for later analyses of BNP and NE concentrations using the respective Enzyme linked Immunosorbent Assay (ELISA) kits (CSB-E17436c, CUSABIO BIOTECH, Wuhan, China) according to the manufacturer’s instructions. The ANG II and Ang (1-7) levels in hypothalamus, and

the NE concentrations in kidney and ventricle were also detected by respective ELISA kits (CSB-E15778c and CSB-EQ027505DO, CUSABIO BIOTECH, Wuhan, China).

References

[1] Chen W, Du H, Lu J, Ling Z, Long Y, Xu Y et al. Renal Artery Vasodilation May Be An Indicator of Successful Sympathetic Nerve Damage During Renal Denervation Procedure. Sci Rep 2016;6:37218.

[2] Lu J, Wang Z, Zhou T, Chen S, Chen W, Du H et al. Selective proximal renal denervation guided by autonomic responses evoked via high-frequency stimulation in a preclinical canine model. Circ Cardiovasc Interv 2015;8.

[3] Wang GD, Cheng LG, Fan RX, Irwin DM, Tang SS, Peng JG et al. Signature of balancing selection at the MC1R gene in Kunming dog populations. PLoS One 2013;8:e55469.

2. Animal Survival

During the course of this study, no dogs were excluded due to renal artery anatomic abnormalities. However, four dogs died accidently for the following reasons: one dog in RDN group died of serious anorexia and diarrhea on the fifth day after ablation; one dog in RDN+HF group died of unexpected ventricular tachycardia and fibrillation during the implantation procedure of ventricular pacing electrode, while another dog in RDN+HF group died of serious infection at the ninth day after intervention; one dog in HF-control group also died of serious infection at the eighteenth day after intervention. Thus, in total, 20 dogs (6 in Sham-operated group, 5 in RDN group, 4 in RDN+HF group, 5 in HF-control group) completed all the research items and were used in data analysis.

Supplemental Table S1. The primer sequences of all genes in this study.

Primer Name	5' to 3'	bp
GAPDH forward	ATTCCACGGCACAGTCAAG	19
GAPDH reverse	TCCACAACATACTCAGCACCA	21
ACE forward	GCGATTGGCTCAGACACCCT	20
ACE reverse	GGTAATTGTTGGGCATCGGTG	21
ACE2 forward	GGTGGGAGATGAAGCGAAAT	20
ACE2 reverse	GACAAAGGGCTTCTTGAAACTG	22
AT1R forward	AAGAACAAGCCAAGAAAGATGA	23
AT1R reverse	GGCGGTGTCAACAATATCTGC	21
Beta-1 AR forward	GCTGCTACAACGACCCCAAGT	21
Beta-1 AR reverse	AACACCCGCAGGTACACGAA	20

ACE=angiotensin-converting enzyme; ACE2=angiotensin-converting enzyme 2; AT1R= angiotensin II type I receptors; Beta-1 AR=β1-adrenergic receptor.

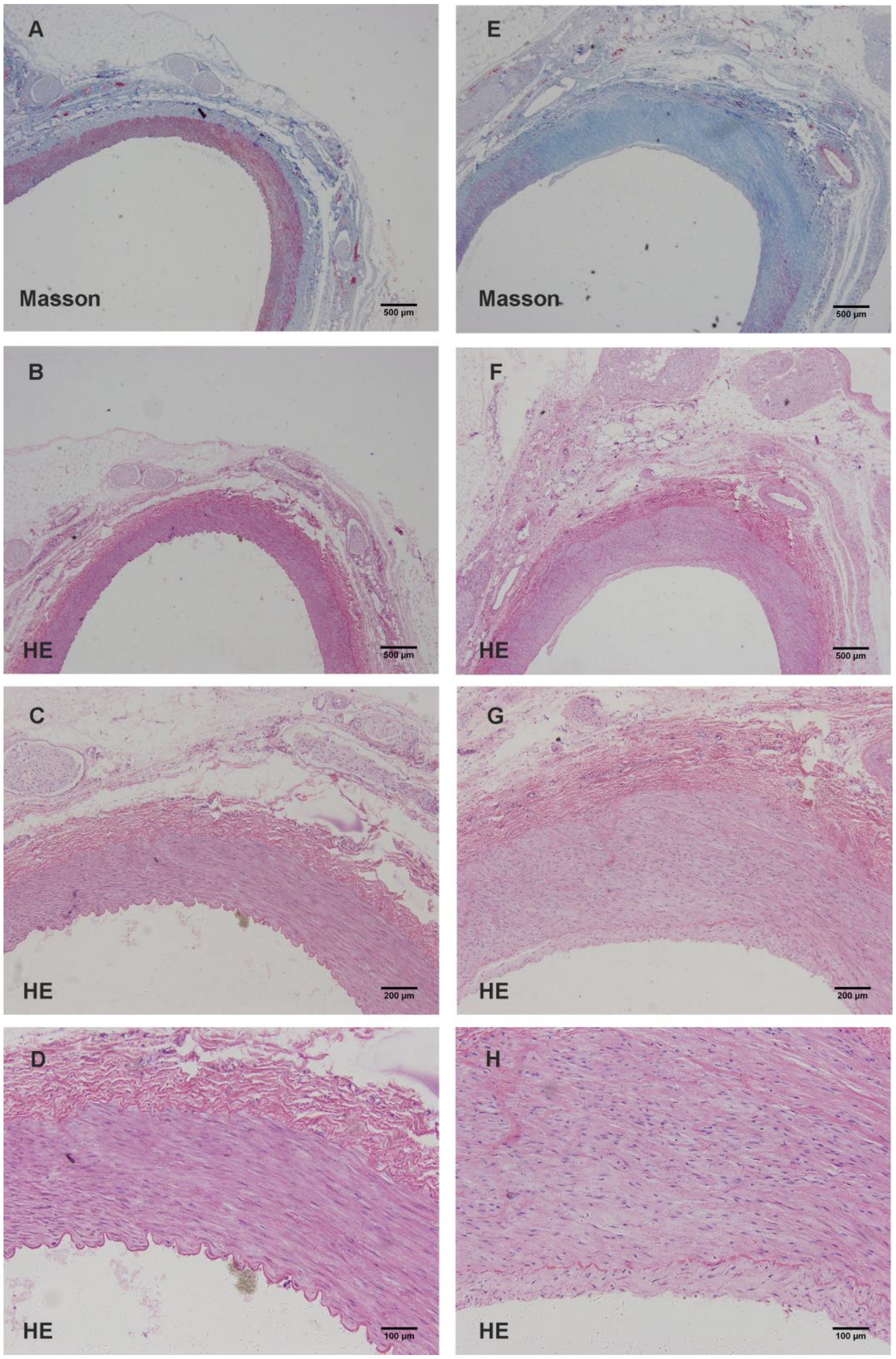

Supplemental Figure S1. Representative hematoxylin-eosin and Masson's staining images of renal artery with ablation lesion area and non-ablation area.

The A and E were representative images of Masson's staining, while the B, C, D, F, G and H were representative images of hematoxylin-eosin staining. By masson's staining, significant hyperplasia of collagen fibers (colored blue) was found in ablation area (E) but not in non-ablation area (A). Through hematoxylin-eosin staining, the wavy line of endothelium disappeared, and even ruptured, and apparent medial hyperplasia was simultaneously found in ablation area (F, G, H) but not in non-ablation area (B, C, D).

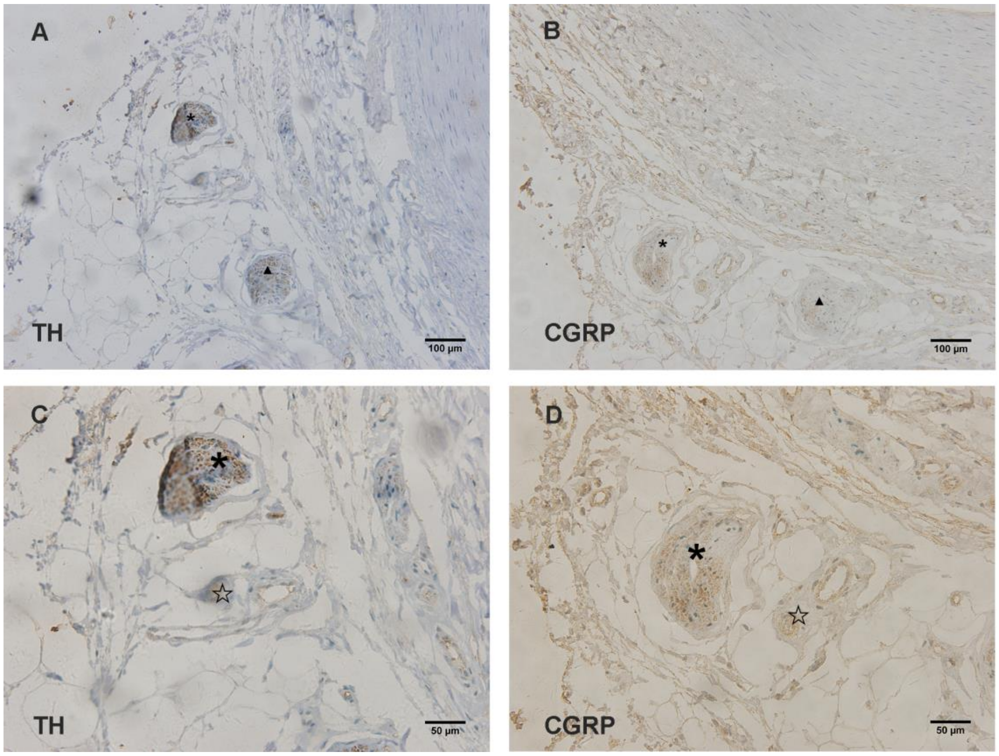

Supplemental Figure S2. Representative immunohistochemical images related to the renal afferent and efferent nerves contained in the same nerve bundle.

A and C were stained with polyclonal antibodies to tyrosine hydroxylase (TH) to label the renal efferent sympathetic nerves; B and D were stained with polyclonal antibodies to calcitonin gene related peptide (CGRP) to label the renal sensory afferent nerves. The same symbol indicated same structure in different images.
